# PEG 200-Assisted Ultrasound Extraction and Characterization of Mulberry Anthocyanins: Optimization, Bioactivities, and Stability

**DOI:** 10.3390/foods15193414

**Published:** 2026-09-24

**Authors:** Nina Bao, Wei Zhao, Qingyang Meng, Lulu Cheng, Marwan M. A. Rashed, Xingtao Zhang, Fangkai Han

**Affiliations:** 1 School of Life Sciences and Food Health, Suzhou University, Suzhou 234000, China; 2 Collaborative Technology Service Center for the High-Value Processing of Green Agricultural Products (Prepared Foods) in the Yangtze River Delta Region, Suzhou University, Suzhou 234000, China; 3 College of Bioengineering, Tianjin University of Science and Technology, Tianjin 300457, China; 4 School of Life Sciences, Anhui Agricultural University, Hefei 230036, China

**Keywords:** mulberry anthocyanins, PEG-UAE 200, ultrasound-assisted extraction, response surface methodology, anthocyanin bioactivity, stability

## Abstract

Mulberry anthocyanins are promising natural pigments and bioactive compounds, but their efficient recovery is limited by processing-induced degradation. In this study, polyethylene glycol (PEG)-assisted ultrasound extraction was investigated for recovering anthocyanins from Morus nigra fruits. Four PEG grades (PEG 200, 300, 400, and 600) were initially screened, after which PEG 200 was selected for process optimization using response surface methodology with a central composite design. The effects of PEG concentration, extraction time, ultrasonic power, and temperature on total monomeric anthocyanin content were evaluated. The optimized conditions were 75% PEG 200, 18 min, 420 W, and 40 °C, yielding 515.78 ± 0.53 mg cyanidin-3-O-glucoside equivalents/100 g dry weight. C18 solid-phase extraction was subsequently used to obtain an anthocyanin-enriched fraction for spectroscopic and mass-spectrometric characterization. FT-IR analysis indicated functional groups consistent with phenolic and glycosidic constituents, while UHPLC-Q-TOF-MS/MS enabled the tentative annotation of five anthocyanin-related compounds. The optimized crude extract exhibited antioxidant, α-amylase and α-glucosidase inhibitory and antibacterial activities under the respective in-vitro assay conditions. Anthocyanin retention was higher under dark and low-temperature conditions than under fluorescent illumination and elevated temperatures. Overall, the results demonstrate the feasibility of PEG 200-assisted ultrasound extraction for recovering anthocyanin-rich mulberry extracts and provide complementary information on their chemical characteristics, in-vitro bioactivities, and stability.

## 1. Introduction

Mulberry (*Morus nigra*) fruits are valued as a source of bioactive compounds and have traditionally been consumed as food and used for medicinal purposes, particularly in Asian regions. Among their major bioactive constituents, anthocyanins are responsible for much of the characteristic dark-purple coloration of ripe mulberry fruits and have attracted considerable interest because of their reported antioxidant, anti-inflammatory, hypoglycemic, and antimicrobial activities [1,2,3,4]. However, anthocyanins are structurally sensitive compounds whose stability can be affected by processing and storage conditions, including temperature, light exposure, and other environmental factors, which can complicate their efficient recovery and subsequent utilization [5,6].

The development of efficient extraction strategies is therefore important for recovering anthocyanins while limiting losses during processing. In recent years, increasing attention has been directed toward extraction approaches that can reduce solvent consumption, extraction time, and energy requirements while maintaining high recovery of target compounds [7,8]. Polyethylene glycol (PEG) has been investigated as a water-miscible polymeric extraction medium for phenolic compounds and other plant-derived constituents [9,10,11]. Its physicochemical properties, including viscosity and molecular size, vary with molecular weight and may consequently influence solvent accessibility and mass transfer during extraction. PEG has also been discussed in the context of solvent recovery and reduced volatility, although the environmental performance of a PEG-based extraction process ultimately depends on the complete process, including solvent concentration, recovery, reuse, and downstream purification [12,13,14,15]. Ultrasonic-assisted extraction (UAE) is another widely investigated approach for recovering bioactive compounds from plant materials. The application of ultrasound can promote cavitation and mechanical effects that facilitate disruption of plant tissues and enhance contact between the extraction medium and intracellular constituents, thereby potentially improving mass transfer and reducing extraction time [16,17]. The efficiency of UAE is influenced by several process variables, including solvent composition, temperature, ultrasound power or amplitude, extraction time, and solid-to-liquid ratio. Consequently, optimization of these variables is necessary to obtain a suitable balance between compound release and stability. For anthocyanin extraction specifically, ultrasound-assisted approaches have been investigated for several fruit and plant matrices, including mulberry. Previous work on Morus nigra has demonstrated that ultrasound-assisted extraction can be optimized by considering solvent composition and temperature together with ultrasound-related parameters, highlighting the importance of process optimization rather than relying on a single extraction variable [18]. However, the extraction behavior of PEG solutions with different molecular weights under ultrasound-assisted conditions has received comparatively limited attention in mulberry anthocyanin extraction. The integration of low-volatility PEG-based solvent systems with ultrasonic-assisted extraction can synergistically optimize extraction conditions, mitigate the limitations inherent to individual extraction methods, and substantially enhance both the yield and bioactivity of anthocyanins [19,20].

The molecular weight of PEG is particularly relevant because it is associated with differences in viscosity and molecular mobility, which may affect solvent penetration and mass transfer within plant tissues [21]. Previous studies have also demonstrated that PEG-based extraction performance can depend strongly on PEG concentration and the physicochemical characteristics of the extraction system [9,10,11]. Accordingly, selection of an appropriate PEG molecular weight should precede optimization of the remaining extraction parameters rather than assuming that a single PEG grade is universally optimal. At the same time, PEG concentration, extraction time, ultrasonic power, and temperature may interact with one another, making optimization based solely on independent single-factor experiments insufficient for identifying the best combination of operating conditions. Response surface methodology (RSM), particularly when implemented using a central composite design (CCD), provides a statistical framework for evaluating linear, quadratic, and interaction effects and for identifying an optimum within a defined experimental region. Such approaches have been successfully applied to optimize ultrasound-assisted anthocyanin extraction from mulberry and other plant materials [18].

Despite these developments, systematic evaluation of PEGs with different molecular weights for ultrasound-assisted extraction of mulberry anthocyanins, followed by multivariable optimization of the selected PEG system, remains limited [9,21,22]. In addition, the chemical characteristics and functional properties of extracts obtained using such a combined extraction strategy require further investigation. The present study therefore considers not only anthocyanin recovery but also the chemical characterization, in-vitro functional properties, and stability of the resulting optimized extract.

Accordingly, the objectives of this study were to: (1) compare PEG 200, PEG 300, PEG 400, and PEG 600 as extraction media for recovering total monomeric anthocyanins from mulberry fruit and select the PEG grade providing the highest total monomeric anthocyanin content (TAC); (2) optimize the selected PEG-assisted ultrasound extraction system, based on PEG 200, using single-factor experiments followed by response surface methodology based on a central composite design, with PEG concentration, extraction time, nominal ultrasonic power, and temperature as independent variables; (3) experimentally validate the model-predicted optimum; (4) characterize an anthocyanin-enriched fraction obtained from the optimized PEG 200 extract using FT-IR spectroscopy and UHPLC-Q-TOF-MS/MS; (5) evaluate the antioxidant, α-amylase and α-glucosidase inhibitory and antibacterial activities of the optimized crude PEG 200 extract; and (6) investigate anthocyanin retention under different light and temperature conditions. Thus, the study was structured as a sequential workflow (Figure 1) in which PEG-grade screening preceded optimization of the selected PEG 200 system, followed by experimental validation and complementary chemical, bioactivity, and stability assessments.

## 2. Materials and Methods

### 2.1. Chemicals and Reagents

Polyethylene glycols (PEG) with nominal molecular weights of 200, 300, 400, and 600 g/mol, respectively, 2,2-diphenyl-1-picrylhydrazyl (DPPH), 2,2′-azinobis (3-ethylbenzothiazoline-6-sulfonic acid) (ABTS), Trolox, and 2,4,6-tris(2-pyridyl)-s-triazine (TPTZ) were procured from Shanghai Macklin Chemistry Co., Ltd. (Shanghai, China). Additional analytical-grade reagents were sourced from Sinopharm Chemical Reagent Co., Ltd. (Shanghai, China).

### 2.2. Materials and Sample Preparation

Mulberry fruits (*Morus nigra*) were obtained from a commercial vegetable market in Suzhou City, Anhui Province, China. The fruits were dried at 40 °C to constant mass, which required approximately 72 h. The dried material was pulverized using an XL-04B Pulverizer (Guangzhou Xulang Machinery Equipment Co., Ltd., Guangzhou, China), passed through a 40-mesh sieve, and stored at 4 °C until extraction.

### 2.3. Polyethylene Glycol 200-Assisted Ultrasonic Extraction (PEG-UAE)

#### 2.3.1. Screening of PEG Molecular Weight

PEG solutions prepared from PEG 200, PEG 300, PEG 400, and PEG 600 were initially evaluated to determine the PEG grade providing the highest total monomeric anthocyanin content. Approximately one g of powdered mulberry sample was extracted with 50 mL of 75% (*v*/*v*) aqueous PEG solution and subjected to ultrasonic treatment at 40 °C for 20 min using a probe-type cell crusher (HX-IID, Shanghai Huxi Industry Co. Ltd., Shanghai, China), operating at a nominal maximum power of 1000 W and frequency of 20 kHz. PEG concentration was expressed throughout the study as % (*v*/*v*). Thus, a 75% (*v*/*v*) PEG solution consisted of 75 mL PEG and sufficient distilled water to obtain a final volume of 100 mL.

PEG 200 was selected as the extraction medium for all subsequent optimization and validation experiments because it provided the highest TAC among the PEG grades evaluated in the preliminary screening.

#### 2.3.2. Determination of Total Monomeric Anthocyanin Content

The TAC of mulberry extracts was determined using the pH differential method described by Huang et al. [21]. which is based on the structural transformation of anthocyanins at different pH values. Absorbance was measured at 520 nm and 700 nm using a UV–Vis spectrophotometer (U-3900H, Hitachi Co., Ltd., Tokyo, Japan). Briefly, one mL of appropriately diluted extract was mixed with 3 mL of potassium chloride buffer (0.025 M, pH 1.0) or 3 mL of sodium acetate buffer (0.4 M, pH 4.5). Samples were equilibrated at room temperature in the dark for 15 min, after which absorbance was measured at 520 nm (maximum absorption) and at 700 nm (to correct for haze and turbidity). The absorbance difference (A) was calculated as:

(1) $$A={\left(A_{520}-A_{700}\right)}_{pH1.0}-{\left(A_{520}-A_{700}\right)}_{pH4.5}$$

The TAC was calculated using cyanidin-3-O-glucoside as the reference compound and expressed as mg cyanidin-3-O-glucoside equivalents (CG)/100 g dry weight (DW) and calculated as follows:

(2) $$Y(mg\;CGE/100\;g\;DW)=\frac{A}{\varepsilon L}\times M_{w}\times DF\times \frac{V}{M_{t}}$$

where (Y) represents TAC expressed as mg cyanidin-3-glucoside equivalents per 100 g dry weight (DW); (A) is the absorbance difference defined in Equation (1); (V) is the extract volume (mL); (DF) is the dilution factor; (M_w_) is the molecular weight of cyanidin-3-glucoside (449.2 g/mol); (ε) is the molar extinction coefficient of cyanidin-3-glucoside (26,900 L/mol/cm); (L) is the path length of the cuvette (1 cm); and (M_t_) is the mass of sample (1 g).

All analytical measurements were performed in triplicate and expressed as mean ± standard deviation (SD).

#### 2.3.3. Experimental Design and Response Surface Methodology

PEG 200 was selected for subsequent optimization based on its higher TAC relative to the other PEG molecular-weight grades evaluated in the preliminary screening. Single-factor experiments were performed to investigate the effects of PEG 200 concentration, extraction time, ultrasonic power, and extraction temperature. The investigated ranges were 20–100% PEG 200, 5–50 min, 200–600 W nominal ultrasonic power, and 20–70 °C, respectively. A liquid-to-solid ratio of 50 mL/g was selected based on preliminary experiments and was subsequently maintained during the optimization experiments. Response surface methodology using a central composite design was then applied to evaluate the effects and interactions of the four independent variables. The factors were coded as follows: A: PEG 200 concentration (% *v*/*v*), B: extraction time (min), C: nominal ultrasonic power (W), and D: extraction temperature (°C).

Thirty experimental runs, including six replicates at the central point, were generated using Design-Expert software version 13. A second-order polynomial model was fitted to the experimental data. Analysis of variance (ANOVA) was used to evaluate model significance and the significance of individual linear, interaction, and quadratic terms.

#### 2.3.4. Model Validation

Model validation was conducted by experimentally evaluating the optimized extraction conditions predicted by Design-Expert software (version 13). The theoretical optimum was rounded to practical experimental settings, and the resulting conditions were evaluated experimentally. The predicted TAC corresponding specifically to the rounded experimental conditions was calculated from the fitted regression model and compared with the experimentally measured TAC. The prediction error was calculated from the difference between the predicted and experimental responses.

### 2.4. C18 Solid-Phase Extraction

The optimized crude PEG 200 extract was subjected to C18 solid-phase extraction to obtain an anthocyanin-enriched fraction for structural characterization and to reduce PEG-related interference during spectroscopic and mass-spectrometric analyses [23]. The C18 cartridge (Model A14-SL02, LeowLab Scientific, Guangzhou, China) was sequentially conditioned with methanol and 0.1% (*v*/*v*) formic acid in water. The crude extract was acidified with 0.1% formic acid before loading onto the cartridge. After sample loading, the cartridge was washed with acidified aqueous solution to remove PEG 200 and other highly polar constituents. Anthocyanin-containing compounds were subsequently eluted with methanol containing 0.1% formic acid. The eluate was concentrated under reduced pressure and freeze-dried using a freeze dryer (LGJ-12N, Yaxingyike, Beijing, China). The C18-SPE procedure was used for analytical enrichment and was not intended as a quantitative preparative purification step. SPE recovery and anthocyanin recovery were not determined in the present study.

### 2.5. SEM Analysis

The mulberry residues were rinsed three times using water, then freeze-dried. Afterward, they were placed on aluminum stubs, coated with a thin layer of gold through sputtering, and analyzed using a scanning electron microscope (Model SU 1510, Hitachi, Tokyo, Japan) [24,25].

### 2.6. FT-IR Characterization

Fourier-transform infrared (FT-IR) spectroscopy was used to characterize functional groups in the C18-SPE anthocyanin-enriched fraction [26]. Purified and lyophilized material was mixed with KBr and pressed into pellets. Spectra were recorded using a Nicolet Summit FT-IR spectrometer (Thermo Scientific, Waltham, MA, USA) over the range of 4000 to 500 cm^−1^.

### 2.7. UHPLC-Q-TOF-MS/MS Analysis

The anthocyanin composition of the C18-SPE anthocyanin-enriched fraction was characterized according to [27], with some modifications, using ultra-high-performance liquid chromatography with quadrupole time-of-flight tandem mass spectrometry (UHPLC-Q-TOF-MS/MS) equipped with a Triple TOF 6600+ system (AB SCIEX, Marlborough, MA, USA). Chromatographic separation was performed on a Kinetex XB-C18 column (2.6 μm, 100 × 2.1 mm; Phenomenex, Torrance, CA, USA). The mobile phase consisted of 0.1% formic acid in water (A) and methanol (B). The gradient elution program was set as follows: 5% solvent B was maintained from 0 to 2 min, followed by a linear increase of solvent B from 5% to 95% between 2 and 28 min. Subsequently, 95% solvent B was maintained for 5 min to wash the column, followed by re-equilibration with 5% solvent B for 5 min before the next injection. The flow rate was maintained at 0.4 mL/min, and the injection volume was 5 μL. Mass spectrometric analysis was conducted in positive electrospray ionization (ESI^+^) mode. The ion spray voltage was set at 5500 V, and the ion source temperature was maintained at 550 °C. The collision energy was set at 35 ± 15 eV with collision energy spread. The MS1 and MS/MS spectra were acquired over an *m*/*z* range of 50–1000. The acquired data were processed using SCIEX OS software (Version 2.0, AB SCIEX, Framingham, MA, USA). Compound assignments were based on precursor-ion accurate mass and characteristic product ions and are therefore reported as tentative annotations because authentic reference standards were not used for definitive identification of all detected compounds.

### 2.8. Antioxidant Activity

The antioxidant activity of the optimized crude PEG 200 extract was evaluated using DPPH, ABTS, FRAP, and reducing-power assays. For all assays, results were expressed as μmol Trolox equivalents per gram of the original dry mulberry material, provided that this is how the current calculations were performed. The DPPH assay was carried out as described by Brand-Williams et al. [28]. Specifically, 2 mL of optimized PEG 200 extracts was mixed with 4 mL of 0.1 mM DPPH solution and incubated in the dark at ambient temperature for 30 min. Subsequently, absorbance was recorded at 517 nm employing a SP-723 spectrophotometer (Shanghai Spectrum Instruments Co., Ltd., Shanghai, China). Antioxidant activity was quantified and expressed as Trolox equivalents (TE) per gram of dry weight (μmol TE/g DW).

The ABTS radical scavenging capacity was determined following the protocol outlined in our previous work [29], with minor modifications. The ABTS stock solution was prepared by combining 10 mL of 2.0 mmol/L ABTS solution with 0.1 mL of 70 mmol/L potassium persulfate, vortexing the mixture, and allowing it to incubate at room temperature for 12 to 16 h. The resultant working ABTS solution was diluted with ethanol to achieve an absorbance of 0.7 ± 0.02 at 734 nm. Trolox standards ranging from 0 to 0.333 mmol/L were employed for calibration, and results were expressed as μmol TE/g DW.

The ferric-reducing antioxidant power (FRAP) assay was performed in accordance with the methodology described by Oldoni et al. [30]. A 0.1 mL aliquot of optimized PEG 200 extracts was combined with 2.9 mL of FRAP reagent, which consisted of acetate buffer, TPTZ solution, and FeCl_3_. The mixture was incubated at 37 °C for 30 min, after which absorbance was measured at 593 nm. Trolox standards (0–0.5 mmol/L) served as the reference, and antioxidant activity was expressed as μmol TE/g DW.

The reducing antioxidant (RA) assay was conducted based on the procedure reported by Bao et al. [31], with slight modifications. Briefly, 200 µL of the sample was mixed with 0.5 mL of phosphate buffer (200 mmol/L, pH 6.6) and 0.5 mL of 1.0% (*w*/*v*) potassium ferricyanide solution. The mixture was vortexed and incubated at 50 °C for 30 min. Subsequently, 0.5 mL of 10.0% (*w*/*v*) trichloroacetic acid was added, and the sample was centrifuged at 2500× *g* for 10 min. Then, 1.2 mL of the supernatant was transferred to a new tube and combined with 0.12 mL of 0.1% (*w*/*v*) ferric chloride solution. Absorbance was measured at 700 nm using a Tecan Spark spectrophotometer (Tecan, Shanghai, China).

### 2.9. In Vitro Enzyme Inhibitory Activities

#### 2.9.1. α-Amylase Inhibitory Activity

The α-amylase inhibitory activity of the optimized PEG 200 extract was evaluated following our previously established protocol [29], with minor modifications. In brief, 50 μL of the optimized PEG 200 extract was combined with 50 μL of α-amylase solution (3 U/mL) and incubated at 37 °C for 20 min. Subsequently, 100 μL of soluble starch solution (5 mg/mL) was added to the mixture, which was then incubated at 37 °C for an additional 3 min. The enzymatic reaction was terminated by heating the mixture in a water bath at 90 °C for 1 min. Thereafter, 100 μL of 3,5-dinitrosalicylic acid (DNS) reagent was introduced, and the reaction mixture was incubated at 90 °C for 10 min. Following cooling, 900 μL of distilled water was added, and the absorbance was recorded at 540 nm using a Tecan Spark spectrophotometer (Shanghai, China).

#### 2.9.2. α-Glucosidase Inhibitory Activity

The α-glucosidase inhibitory activity of the optimized PEG 200 extract was also assessed following our previously established protocol [29], with minor modifications. In brief, 20 μL of the optimized PEG 200 extract was combined with 20 μL of α-glucosidase solution (3.38 U/mL). This mixture was subsequently added to 120 μL of phosphate-buffered saline (PBS; 0.1 mol/L, pH 6.8) and incubated at 37 °C for 10 min. Thereafter, 20 μL of p-nitrophenyl-α-D-glucopyranoside (pNPG; 2.5 mmol/L) was added, and the reaction mixture was further incubated at 37 °C for 20 min. The enzymatic reaction was terminated by the addition of 80 μL of sodium carbonate solution (0.2 mmol/L), and the absorbance was recorded at 405 nm using a Tecan Spark spectrophotometer (Shanghai, China).

Acarbose was used as a positive control at concentrations of 10, 50, 100, 150, and 200 μg/mL.

### 2.10. In Vitro Antibacterial Activities

The antibacterial activity of the optimized PEG 200 extract was evaluated against two Gram-positive species, *Staphylococcus aureus* and *Bacillus subtilis*, and one Gram-negative species, *Escherichia coli*, using the Oxford cup method [32]. Bacterial suspensions were adjusted to approximately 1.0 × 10^7^ CFU/mL and inoculated into soft agar. After solidification, Oxford cups containing 200 μL of the optimized crude PEG 200 extract were placed on the agar surface, with 75% PEG 200 (200 μL) as the negative control. For each bacterial strain, three plates were prepared, and the inhibition zones were measured after incubation for 24 h at 37 °C. Results were expressed as mean ± standard deviation (*n* = 3).

### 2.11. Light Stability Assessment

The optimized PEG 200 extract was stored under fluorescent illumination and dark conditions at room temperature. Samples were evaluated for TAC at 0, 1, 2, 3, 4, and 5 days. The fluorescent light source was located approximately 18 inches from the samples. The electrical specifications of the lamp were recorded as 220 V, 50 Hz, and 0.37 A. However, spectral irradiance or illuminance at the sample surface was not independently measured.

### 2.12. Temperature Stability Assessment

The thermal stability of the optimized PEG 200 extract was assessed at temperatures of 4, 25, 40, 60, 80, and 100 °C under dark conditions. Specifically, 0.5 mL of the optimized PEG 200 extract was placed into a 10 mL volumetric flask and diluted to volume with a buffer solution (pH = 1). The samples were then sealed and stored at the specified temperatures. Aliquots were collected at two-hour intervals over a total duration of 10 h, and the TAC was subsequently measured.

### 2.13. Statistical Analysis

Data were expressed as mean ± standard deviation. One-way ANOVA followed by Tukey’s multiple-comparison test was used to assess differences among groups, with *p* < 0.05 considered statistically significant. For the response surface experiment, the CCD comprised 30 experimental runs, including six independently performed center-point experiments. The replicated center points were used to estimate pure experimental error and to assess model lack-of-fit. The quadratic model was evaluated using analysis of variance and model adequacy statistics, including *R*^2^, adjusted *R*^2^, predicted *R*^2^, coefficient of variation, adequate precision, and lack-of-fit. Analytical measurements were performed in triplicate; these analytical replicates were distinguished from independent extraction experiments.

## 3. Results and Discussion

### 3.1. Determination of the Wavelength for the pH Differential Method

As shown in Figure 2, the mulberry extract exhibited a prominent absorption maximum at 520 nm under pH 1.0 conditions. Because anthocyanins undergo structural and color changes as a function of pH, whereas many non-anthocyanin constituents exhibit comparatively smaller changes in absorbance, the pH differential method can be used to estimate total monomeric anthocyanin content [33]. The absorbance difference between the two pH conditions was greatest at 520 nm; therefore, 520 nm was selected for determination of total monomeric anthocyanin content (TAC).

### 3.2. Selection of PEG Solutions

Polyethylene glycol (PEG) solutions of different molecular weights (PEG 200, PEG 300, PEG 400, and PEG 600) were evaluated for their ability to extract anthocyanins from mulberry fruit. PEG contains oxygen-containing functional groups that can participate in hydrogen-bonding interactions with hydroxyl-containing compounds, which may contribute to the solubilization of phenolic constituents [11]. As shown in Figure 3A, PEG molecular weight significantly affected total anthocyanin content. Among the tested PEG grades, PEG 200 produced the highest TAC, approximately 483 mg CGE/100 g DW, which was significantly higher than those obtained with PEG 300, PEG 400, and PEG 600 (*p* < 0.05). A progressive decrease in extraction response was observed with increasing PEG molecular weight. The higher extraction response obtained with PEG 200 may be associated with differences in the physicochemical properties of the extraction media. In particular, lower-molecular-weight PEG solutions generally exhibit lower viscosity and greater molecular mobility, which may facilitate solvent penetration and mass transfer within plant tissues. Conversely, the higher viscosity and larger molecular size associated with higher-molecular-weight PEGs may increase resistance to solvent movement and reduce accessibility to intracellular constituents. This interpretation is consistent with previous observations concerning the influence of PEG molecular characteristics on extraction performance [21,34].

SEM observations provided complementary evidence of differences in the morphology of the extracted mulberry residues. The PEG 200-treated residue exhibited a more disrupted surface, with pores, cracks, and irregular fractures, whereas the residues obtained using PEG 400 and PEG 600 appeared comparatively compact and ordered (Figure 4). These morphological differences are consistent with greater disruption of the plant matrix under the PEG 200 condition and may help explain the higher anthocyanin recovery observed with this PEG grade. However, SEM provides morphological evidence rather than a direct measurement of solvent penetration or mass transfer.

Ultrasound-assisted extraction involves acoustic cavitation, including bubble collapse, microjet formation, shock waves, and localized shear forces, which promote cell wall disruption and enhance mass transfer [35,36]. The viscosity of the extraction medium can also influence ultrasound propagation and cavitation behavior; highly viscous media may attenuate ultrasonic effects and reduce mass-transfer efficiency [37]. Thus, the lower extraction response observed with PEG 300–600 may be partly associated with their higher viscosity and the resulting changes in mass transfer and ultrasound propagation. Because viscosity and acoustic cavitation intensity were not directly measured in the present study, however, this explanation should be considered a plausible interpretation rather than a directly demonstrated mechanism.

In summary, the results demonstrate that PEG molecular weight was an important factor affecting anthocyanin recovery under the conditions tested, with PEG 200 providing the highest extraction response. PEG 200 was therefore selected for subsequent optimization and characterization.

Based on the preliminary PEG-grade screening, PEG 200 produced the highest TAC and was therefore selected as the extraction medium for all subsequent optimization and validation experiments. The optimization stage was consequently performed exclusively using PEG 200, with PEG concentration, extraction time, ultrasonic power, and temperature investigated as the principal process variables.

### 3.3. Optimization and Validation of PEG 200-Assisted Ultrasound Extraction

#### 3.3.1. Single-Factor Experiments

The effects of PEG 200 concentration, ultrasonic time, ultrasonic power, and extraction temperature on anthocyanin extraction were initially investigated using single-factor experiments. The liquid-to-solid ratio was also examined during the preliminary experiments but was not included as a factor in the subsequent response surface design. Increasing PEG 200 concentration resulted in an increase in TAC, reaching 497.90 ± 17.10 mg CGE/100 g DW at 80% PEG 200. Further increasing the concentration to 100% resulted in a marked decline to 204.92 ± 11.39 mg CGE/100 g DW (Figure 3B). Similarly, the highest responses observed in the individual-factor experiments were 501.50 ± 20.33 mg CGE/100 g DW at 20 min, 506.99 ± 31.70 mg CGE/100 g DW at 400 W, and 506.40 ± 4.73 mg CGE/100 g DW at 40 °C (Figure 3C–E). Based on these preliminary results, the central levels for the central composite design (CCD) were established as 80% PEG 200, 20 min, 400 W, and 40 °C.

These results indicate that the response did not increase linearly with any of the investigated variables. Instead, each factor exhibited a region in which further increases were associated with diminishing or declining anthocyanin recovery. This behavior justified the subsequent use of a multivariable response-surface approach.

#### 3.3.2. Response Surface Methodology and Optimum Condition

The CCD comprising 30 experimental runs, including six center-point replicates, was used to investigate the combined effects of PEG 200 concentration (A), ultrasonic time (B), ultrasonic power (C), and extraction temperature (D) on TAC. A total of 30 experimental trials, including six replicates at the central point, were performed as detailed in Table 1. The significance of the model and individual variables was assessed via ANOVA based on the fitted quadratic model, as presented in Table 2. The experimental TAC values ranged from 324.92 to 516.49 mg CGE/100 g DW. The fitted quadratic model was highly significant (*p* < 0.0001), indicating that the model adequately described the relationship between the investigated extraction variables and the TAC response within the experimental design space. The fitted equation was:

(3) $$Y=513.54-10.00A+0.3879B+7.17C+3.27D+20.26AB-5.95AC+15.17AD-19.98BC-10.82BD+16.42CD-16.65A^{2}-33.38B^{2}-34.75C^{2}-34.57D^{2}$$

where *Y* represents TAC and A, B, C, and D represent the coded values of PEG 200 concentration, ultrasonic time, nominal ultrasonic power, and extraction temperature, respectively.

The model predicted an optimum at 75.58% PEG 200, 18.06 min, 420.36 W nominal ultrasonic power, and 40.29 °C, with a predicted TAC of 516.49 mg CGE/100 g DW. For practical validation, these conditions were rounded to 75% PEG 200, 18 min, 420 W, and 40 °C.

The coefficient of determination (*R*^2^ = 0.9984) indicated an excellent fit of the developed quadratic model to the experimental data. Although the model exhibited a high *R*^2^ value, model adequacy was evaluated using multiple statistical parameters, including the lack-of-fit test, coefficient of variation (CV), and adequate precision, rather than relying solely on *R*^2^. The lack-of-fit was not significant (*p* = 0.4074), confirming that the model adequately represents the experimental data without systematic deviation. The low coefficient of variation (CV = 0.80%) demonstrated high reproducibility and low dispersion among experimental results, indicating strong experimental reliability. In addition, the adequate precision value (77.419) indicated a very high signal-to-noise ratio, confirming that the model can be effectively used to navigate the design space. Furthermore, the regression model exhibited a high *F*-value (651.65, *p* < 0.0001), indicating overall significance. The linear terms (A, C, and D), interaction terms (AB, AC, AD, BC, BD, and CD), and quadratic terms (A^2^, B^2^, C^2^, and D^2^) were all statistically significant (*p* < 0.05), indicating that both individual factors and their interactions strongly influenced anthocyanin extraction efficiency. The model statistics and non-significant lack-of-fit support the adequacy of the fitted quadratic model for describing TAC within the investigated experimental domain. Experimental validation under the rounded optimum conditions is presented in Section 3.3.3.

The physicochemical properties of the PEG 200 solution, including polarity and viscosity, likely contribute to variations in the stability and mass transfer of bioactive compounds, consistent with the principle of “like dissolves like.” Specifically, an increase in PEG 200 concentration from 70% to 80% corresponded with an increase in TAC, as depicted in Figure 5A–C. However, further increases beyond this concentration resulted in a decline in TAC values. These observations align with the findings of Huang et al. [21] and may be attributed to alterations in PEG polarity; when the polarity of PEG closely matches that of the bioactive compounds, extraction efficiency is enhanced, whereas a polarity mismatch leads to reduced extraction, as supported by Zhang and Wang [34]. This phenomenon is reflected in the negative quadratic regression coefficient reported in Table 2.

Ultrasound treatment (Figure 5A,D,E) was demonstrated to facilitate plant tissue swelling and hydration, thereby improving the accessibility of PEG 200 to target compounds. The cavitation-induced collapse of microbubbles generates microjets that disrupt cell walls, promoting the release of intracellular constituents, such as anthocyanins, into the surrounding PEG 200 medium [38]. Nonetheless, prolonged ultrasound exposure can accelerate the degradation of anthocyanins, which are sensitive to thermal and pressure-induced stress [39]. This observation is consistent with Fu et al., who reported similar effects during phenolic compound extraction from *Carya cathayensis* peels.

Regarding temperature effects, increasing the extraction temperature from 30 °C to 40 °C significantly enhanced TAC yield, reaching a maximum at 40 °C, beyond which a notable decline was observed (Figure 5C,E,F). This trend may be explained by increased permeability of plant cell membranes at elevated temperatures, which facilitates solvent penetration and the solubilization of bioactive compounds [40]. However, excessively high temperatures can degrade thermolabile anthocyanins, as reported by Cao et al. [41], and corroborated by the present study.

Ultrasonic power (Figure 5A,D,E) similarly influenced TAC yield by modulating cavitation intensity. TAC increased with ultrasonic power from 300 W to 400 W, corresponding to optimal cavitation conditions at 400 W. Conversely, a sharp decline in TAC was observed at higher ultrasonic powers, likely due to the generation of extreme localized temperatures and pressures that promote degradation of bioactive compounds, including anthocyanins and phenolics [42,43].

Because PEG 200 was used as the extraction medium, potential matrix effects associated with the extraction solvent should be considered when interpreting the spectrophotometric TAC values. In the present study, the extracts were appropriately diluted before the pH-differential measurement, and absorbance at 700 nm was used to correct for haze and turbidity. The TAC values are primarily interpreted as comparative measurements for optimization within the investigated experimental domain.

#### 3.3.3. Validation Process

The quadratic model predicted a theoretical optimum of 75.58% PEG 200, 18.06 min, 420.36 W, and 40.29 °C, corresponding to a predicted TAC of 516.49 mg CE/100 g DW. For practical experimental validation, these conditions were rounded to 75% PEG 200, 18 min, 420 W, and 40 °C. The fitted quadratic regression model was therefore used to calculate the predicted response specifically at these rounded experimental conditions, yielding a predicted TAC of 516.43 mg CE/100 g DW. Under the same rounded conditions, the experimentally determined TAC was 515.78 ± 0.53 mg CE/100 g DW. The absolute difference between the predicted and experimental values was 0.65 mg CE/100 g DW, corresponding to a prediction error of approximately 0.13%. The close agreement between the recalculated predicted and experimental responses supports the adequacy of the model for predicting TAC within the investigated experimental domain [44].

### 3.4. Structural Characterization of the Purified Anthocyanin Fraction

#### 3.4.1. C18-SPE Purification

C18-solid-phase extraction (C18-SPE) was applied to the optimized PEG 200 extract to reduce interference from PEG 200 and other co-extracted components and to obtain an anthocyanin-enriched fraction suitable for structural characterization. The procedure was based on interactions between anthocyanin chromophores and the C18 stationary phase, followed by elution with acidified methanol. The C18-SPE fraction was subsequently used for FT-IR and UHPLC-Q-TOF-MS/MS analyses. It is important, however, to distinguish this analytically enriched fraction from the optimized crude PEG 200 extract used for the functional assays below.

#### 3.4.2. FT-IR Characterization of the Purified Anthocyanin-Rich Fraction

FT-IR spectroscopy was performed to elucidate the structural characteristics of the purified anthocyanin extract obtained through PEG 200-assisted extraction followed by C18-SPE purification. As shown in Figure 6, the purified extract exhibited several characteristic absorption bands associated with anthocyanin glycosides. The broad band centered at 3266 cm^−1^ was assigned to O–H stretching vibrations, consistent with hydroxyl groups in phenolic and carbohydrate-containing constituents [45,46]. The band at 2928 cm^−1^ was associated with C–H stretching vibrations, while the band at 1604 cm^−1^ was assigned to aromatic C=C stretching within conjugated phenolic structures [47]. The bands near 1249 and 1026 cm^−1^ were consistent with C–O–C and C–O stretching vibrations associated with glycosidic and carbohydrate-containing structures in the fraction [48,49]. These spectral features are consistent with the presence of glycosylated phenolic constituents, including anthocyanin-related compounds, in the enriched fraction. The interpretation is further compatible with previous reports describing cyanidin-based glycosides as important mulberry anthocyanins [50,51]. The spectrum was also examined for evidence of residual PEG 200. PEG exhibits C–O–C stretching bands in the approximately 1050–1150 cm^−1^ region. No additional prominent bands attributable specifically to PEG were observed, while the aromatic and glycosidic signals remained prominent. This observation is consistent with substantial reduction in PEG during C18-SPE, as previously discussed by Xue et al. [51], but FT-IR alone does not provide a quantitative measurement of residual PEG. Thus, the FT-IR results provide supporting spectroscopic evidence for an anthocyanin/phenolic-enriched fraction rather than definitive structural identification of individual anthocyanins.

### 3.5. UHPLC-Q-TOF-MS/MS Characterization of Anthocyanins

UHPLC-Q-TOF-MS/MS was used to characterize the anthocyanin profile of the C18-SPE-enriched fraction, and the identified compounds are summarized in Table 3. Peak 1 exhibited a precursor ion at *m/z* 757 and product ions at *m*/*z* 595, 449, and 287. The sequential neutral losses of 162, 146, and 162 Da were consistent with losses of hexose, deoxyhexose, and hexose residues, respectively. Peak 1 was therefore tentatively identified as cyanidin hexose-deoxyhexose-hexoside. Peak 2 exhibited a precursor ion at *m*/*z* 611 and product ions at *m*/*z* 449 and 287, consistent with sequential losses of two hexose residues. It was therefore tentatively identified as cyanidin dihexoside, in agreement with previous observations reported by Kim and Lee [52]. Peaks 3 and 4 showed precursor ions at *m*/*z* 449 and 595, respectively, and both generated a characteristic fragment ion at *m*/*z* 287. The formation of the *m*/*z* 287 fragment was attributed to the loss of a hexose moiety (162 Da) from Peak 3 and a rutinoside moiety (308 Da) from Peak 4. Accordingly, Peaks 3 and 4 were tentatively identified as cyanidin-3-O-glucoside and cyanidin-3-O-rutinoside, respectively, consistent with previous reports [52,53,54,55]. Peak 5 exhibited a precursor ion at *m*/*z* 433 and a characteristic fragment ion at *m*/*z* 271, corresponding to the pelargonidin aglycone following the neutral loss of a hexose moiety (162 Da). Therefore, Peak 5 was tentatively identified as pelargonidin-3-O-glucoside, consistent with previous reports [52,53,54,55].

It should be noted that the bioactivity assays described below were conducted using the optimized crude PEG 200 extract rather than the C18 SPE-enriched fraction. Thus, the observed functional properties represent the activity of the crude optimized extraction product containing PEG 200 and co-extracted constituents, whereas the C18-SPE fraction was used specifically for complementary chemical characterization.

### 3.6. In Vitro Antioxidant Activities

The antioxidant potential of the optimized crude PEG 200 extract was evaluated using DPPH, ABTS, FRAP, and RA assays. The extract exhibited considerable antioxidant activity in all four assays, with values of 187.29 ± 4.42 μmol TE/g DW, 379.94 ± 3.19 μmol TE/g DW, 326.19 ± 7.94 μmol TE/g DW, and 2093.98 ± 74.79 μmol TE/g DW, respectively. These results indicate that the optimized extract possessed substantial radical-scavenging and reducing activity under the respective assay conditions. Because DPPH, ABTS, FRAP, and reducing-power assays rely on different reaction principles, their numerical values should not be interpreted as interchangeable measures of a single antioxidant property.

The antioxidant capacities observed in the present study were comparable to or higher than those reported previously [52,56]. Differences among studies may be attributed to variations in mulberry cultivar, fruit maturity, extraction solvents and conditions, anthocyanin composition, and assay protocols. The strong antioxidant performance may be associated with the efficient recovery of anthocyanins and other phenolic constituents during PEG 200-assisted extraction.

### 3.7. In Vitro α-Amylase and α-Glucosidase Inhibitory Activities

The optimized PEG 200 extract was evaluated for its in vitro inhibitory activity against α-amylase and α-glucosidase under the experimental conditions used, with acarbose serving as the positive control. The assays were performed to characterize the functional properties of the anthocyanin-rich extract and to explore its potential application as a multifunctional food ingredient, rather than to establish physiological or therapeutic effects. The results showed that the optimized PEG 200 extract exhibited inhibitory activity against both enzymes, although its potency differed from that of acarbose. Specifically, the extract showed a lower inhibitory effect on α-amylase than acarbose, yet it elicited a measurable inhibitory response at the concentrations tested. Additionally, the extract exhibited concentration-dependent inhibition of α-glucosidase, with inhibitory strength varying relative to acarbose. These inhibitory effects may be associated, at least in part, with the anthocyanin profile of the extract, including cyanidin-3-O-glucoside and cyanidin-3-O-rutinoside, which were among the anthocyanins tentatively identified via UHPLC-Q-TOF-MS/MS analysis. However, given the complexity of the extract used in the current experiments, the specific contributions of individual anthocyanins to the observed enzyme inhibition remain indeterminate. Therefore, the present results provide evidence of in vitro α-amylase and α-glucosidase inhibitory activity. From a food science perspective, the observed inhibition of α-amylase and α-glucosidase further supports the functional potential of the optimized mulberry extract beyond its antioxidant capacity. Together with its antioxidant and antibacterial properties, these activities support the potential application of the optimized extract as a multifunctional food ingredient in the development of anthocyanin-enriched functional foods and beverages [46,57].

Overall, the observed concentration-dependent inhibition indicates that constituents present in the optimized crude extract can interfere with the catalytic activity of these enzymes in vitro biochemical systems.

### 3.8. Antibacterial Activity

The optimized PEG 200 extract exhibited inhibition against the three tested foodborne bacteria under the experimental conditions used. The inhibition zones were approximately 19 mm for *B. subtilis*, 18 mm for *S. aureus*, and 12 mm for *E. coli* (Table 4). The stronger inhibition observed against the two Gram-positive bacteria than against *E. coli* may be related to differences in bacterial cell envelope structure. Gram-positive bacteria, such as *S. aureus* and *B. subtilis*, lack an outer membrane, whereas *E. coli*, a Gram-negative bacterium, has a thin peptidoglycan layer located between the inner and outer membranes, with the outer membrane acting as an additional barrier to the penetration of antimicrobial compounds [58,59]. This structural difference may contribute to the greater sensitivity of the Gram-positive bacteria observed in the present study. Similar antibacterial activity of mulberry-derived extracts, particularly against Gram-positive bacteria, has also been reported previously [60]. However, no positive antimicrobial control was included in the present assay; therefore, these results should be regarded as preliminary evidence of antibacterial activity under the tested conditions.

### 3.9. Stability of Mulberry Anthocyanins Under Light and Temperature Treatments

#### 3.9.1. Effect of Light Exposure on Anthocyanin Stability

The influence of light exposure on the stability of anthocyanins during storage was investigated, with retention rates under fluorescent light and dark conditions monitored over a five-day period, as illustrated in Figure 7A. Throughout the storage duration, anthocyanin retention exhibited a gradual decline in both conditions. However, the degradation rate was significantly greater under fluorescent light. Notably, after five days, the retention rate in samples exposed to fluorescent light decreased to approximately 73%, whereas those stored in darkness retained a substantially higher level of about 88%. These findings clearly indicate that fluorescent light markedly accelerates the degradation of mulberry anthocyanins, while storage in darkness effectively preserves their stability. This observation aligns with the results reported by Sariya et al. [61] and corroborates the established photosensitivity of anthocyanins, which are susceptible to photo-oxidation and structural modifications upon exposure to ultraviolet and visible radiation [62].

#### 3.9.2. Effect of Temperature on Anthocyanin Stability

Anthocyanin retention was evaluated at 4, 25, 40, 60, 80, and 100 °C for 10 h (Figure 7B). Retention decreased progressively with increasing storage time at all temperatures. At 4 °C, more than 90% of the initial anthocyanin content remained after 10 h. Retention decreased to approximately 85% and 75% at 25 and 40 °C, respectively. At higher temperatures, degradation proceeded more rapidly: less than 40% remained after 2 h at 100 °C, while retention decreased below 50% after 4 h at 80 °C. At 60 °C, approximately 60% remained after 10 h.

Anthocyanins, naturally occurring flavonoids responsible for the red, purple, and blue pigmentation in numerous fruits and vegetables, exhibit chemical stability that is highly influenced by temperature, pH, and storage duration. The present findings clearly demonstrate that elevated temperatures accelerate anthocyanin degradation, primarily due to thermally induced ring-opening reactions leading to chalcone formation, followed by oxidative or hydrolytic breakdown [5,6,63]. At 4 °C, reduced molecular motion and enzymatic activity effectively preserve anthocyanin integrity, whereas at 100 °C, rapid degradation is likely attributable to combined thermal decomposition and enhanced oxidation. These results are consistent with prior research on anthocyanin stability in both model systems and food matrices. For example, Patras et al. [64] reported significant reductions in anthocyanin retention within minutes of thermal processing at temperatures exceeding 80 °C. Taken together, the present results demonstrate that low-temperature and dark storage conditions are more favorable for preserving anthocyanin content in the optimized extract over the tested storage periods.

From a food-processing perspective, the optimized PEG 200-assisted ultrasound extraction provides a defined laboratory-scale approach for recovering anthocyanin-rich mulberry extracts under the investigated conditions. However, the present study did not evaluate scale-up, solvent recovery, residual PEG, process economics, or performance in a real food matrix. Therefore, the practical and industrial applicability of the optimized process should be considered preliminary. Further studies are required to assess these aspects before broader food-processing applications can be established.

## 4. Conclusions

This study evaluated PEG-assisted ultrasound extraction of anthocyanins from mulberry fruit and demonstrated that PEG molecular weight influenced the recovery of total monomeric anthocyanins. Among the PEG grades investigated, PEG 200 produced the highest response and was therefore selected for subsequent optimization. Response surface methodology identified a practical extraction condition of 75% (*v*/*v*) PEG 200, 18 min, 420 W nominal ultrasonic power, and 40 °C. Response surface methodology identified a practical extraction condition of 75% (*v*/*v*) PEG 200, 18 min, 420 W nominal ultrasonic power, and 40 °C. Under these conditions, the experimentally determined TAC was 515.78 ± 0.53 mg CGE/100 g DW, compared with a model-predicted value of 516.43 mg CGE/100 g DW calculated specifically at the rounded experimental conditions, corresponding to a prediction error of approximately 0.13%. C18 solid-phase extraction was subsequently used to obtain an anthocyanin-enriched fraction for chemical characterization. FT-IR spectroscopy provided evidence consistent with hydroxyl-, aromatic-, and glycoside-containing constituents, while UHPLC-Q-TOF-MS/MS enabled the tentative annotation of five anthocyanin-related compounds, including cyanidin- and pelargonidin-based glycosides. Because authentic standards were not used for definitive identification, these assignments should be regarded as tentative. The optimized crude PEG 200 extract exhibited antioxidant, α-amylase and α-glucosidase inhibitory and antibacterial activities under the respective in-vitro assay conditions. These activities should be considered properties of the crude extract, and the present study does not establish the contribution of individual anthocyanins to the observed responses. Anthocyanin retention was also affected by storage conditions, with greater retention under dark and low-temperature conditions than under fluorescent illumination or elevated temperatures. Overall, PEG 200-assisted ultrasound extraction provided an effective approach for recovering anthocyanin-rich mulberry extracts within the investigated experimental domain. Further studies should evaluate solvent recovery and residual PEG, include direct comparison with ultrasound-assisted aqueous extraction, establish compound-specific biological activity, and assess the behavior of the optimized extract in relevant food matrices.

## Figures and Tables

**Figure 1:**
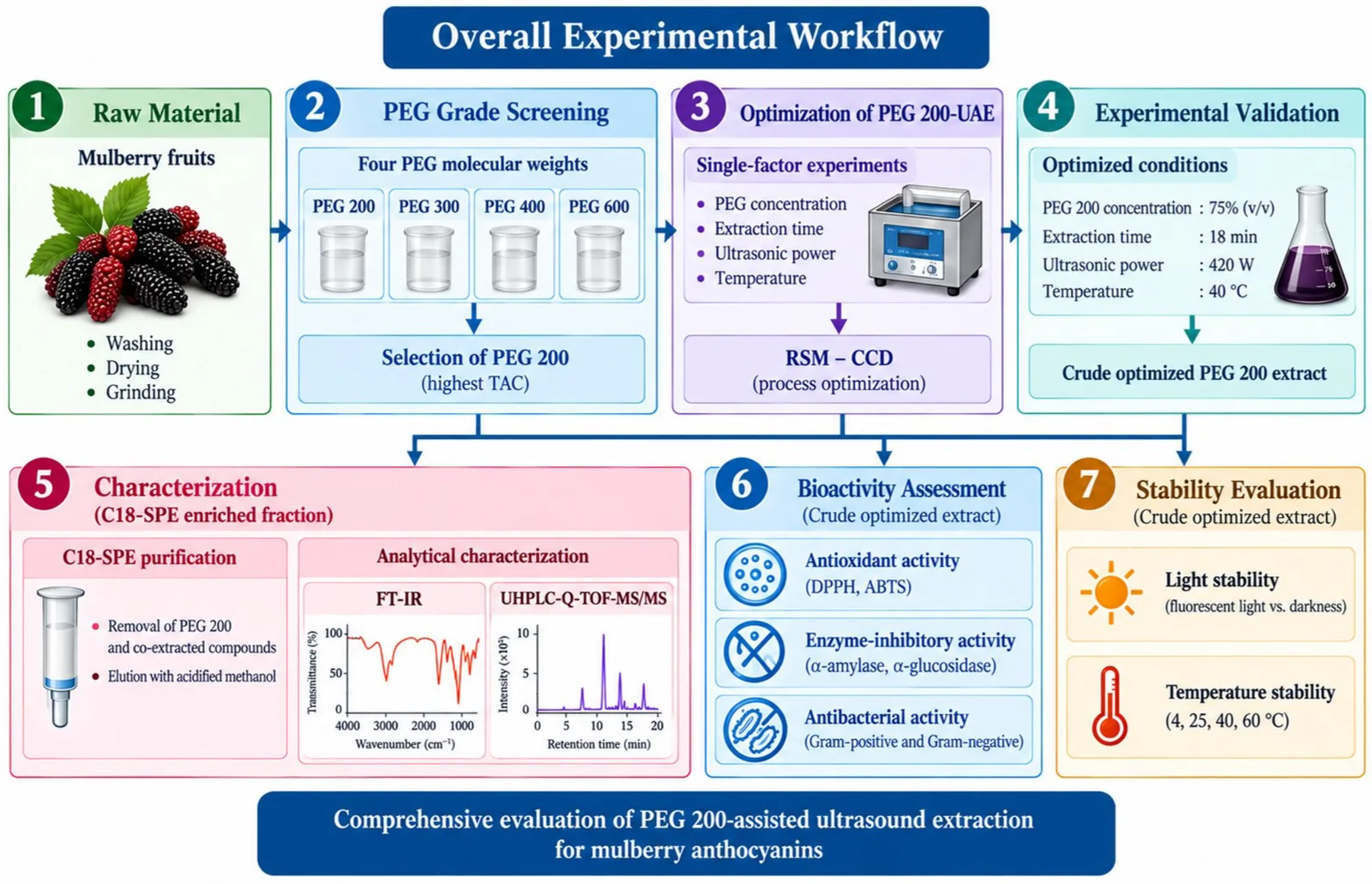
Overall experimental workflow of PEG 200-assisted ultrasound extraction, optimization, validation, and downstream characterization of mulberry anthocyanins. Arrows indicate the sequence and progression of the experimental stages.

**Figure 2:**
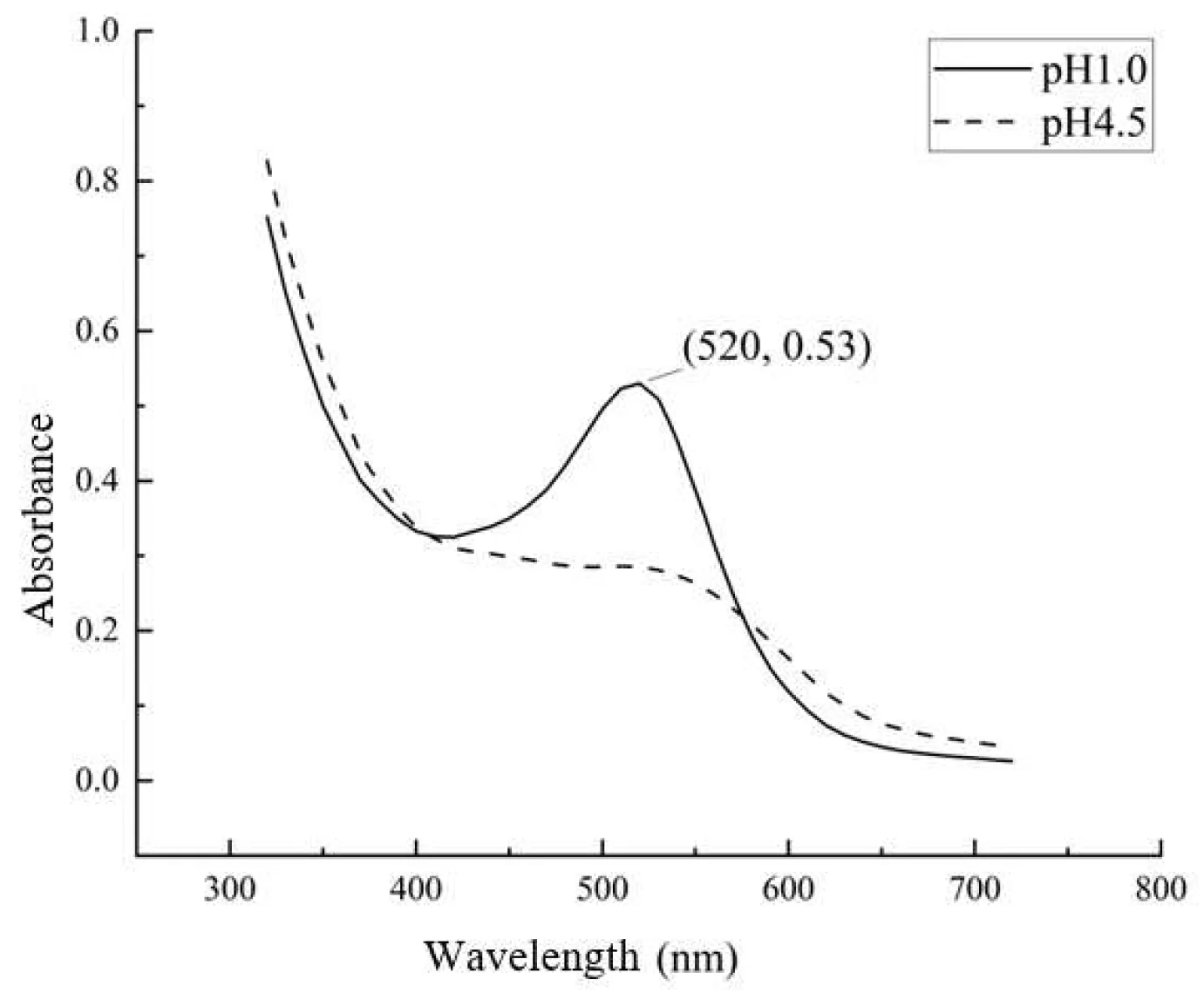
UV-Vis absorption spectrum of mulberry anthocyanin extract.

**Figure 3:**
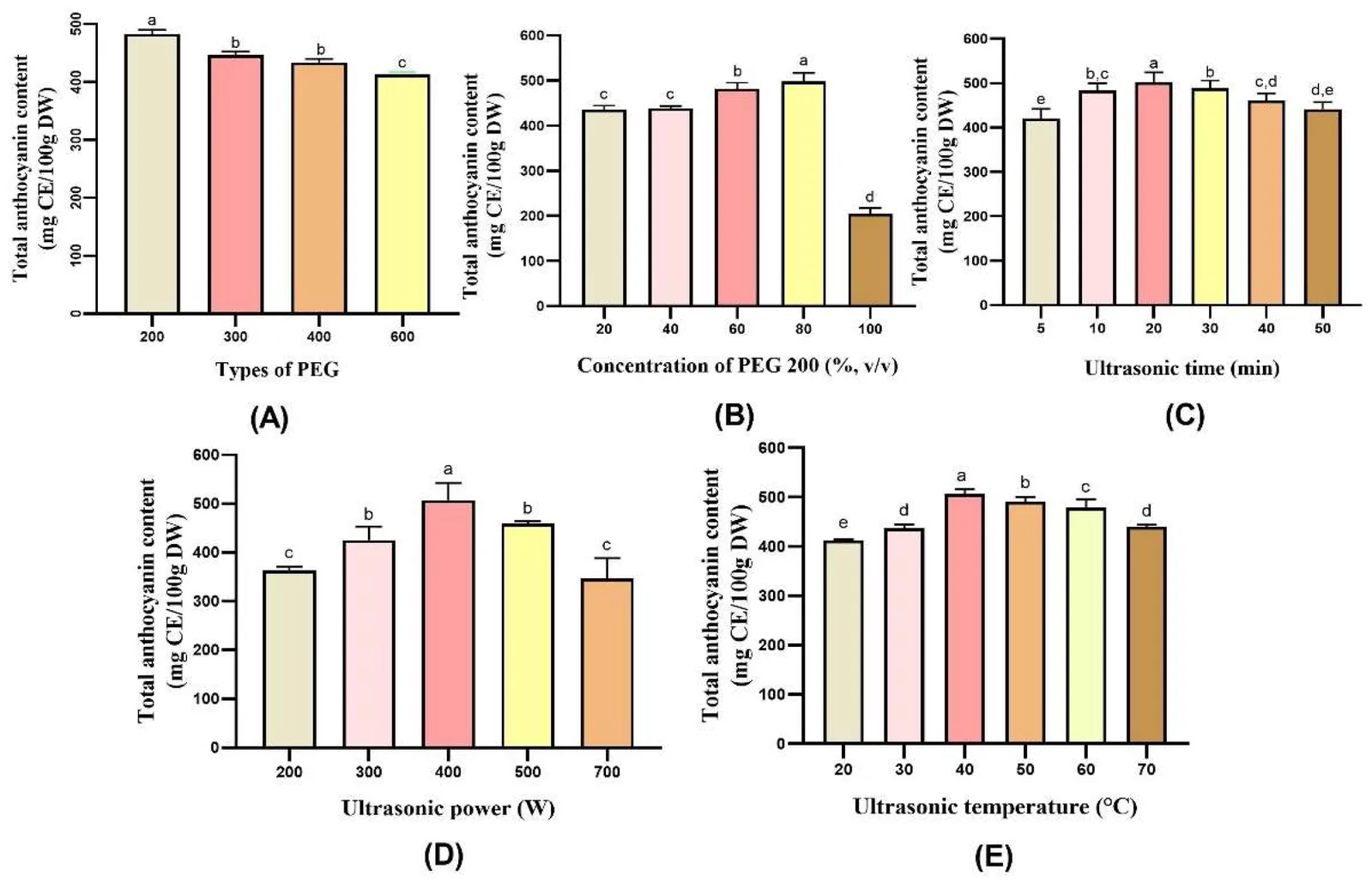
Effect of different types of PEG (**A**), concentration of PEG 200 (**B**), ultrasonic time (**C**), ultrasonic power (**D**), and ultrasonic temperature (**E**) on TAC. Values are presented as means ± SD (*n* = 3). Different letters above the bar showed significant difference at *p* < 0.05.

**Figure 4:**
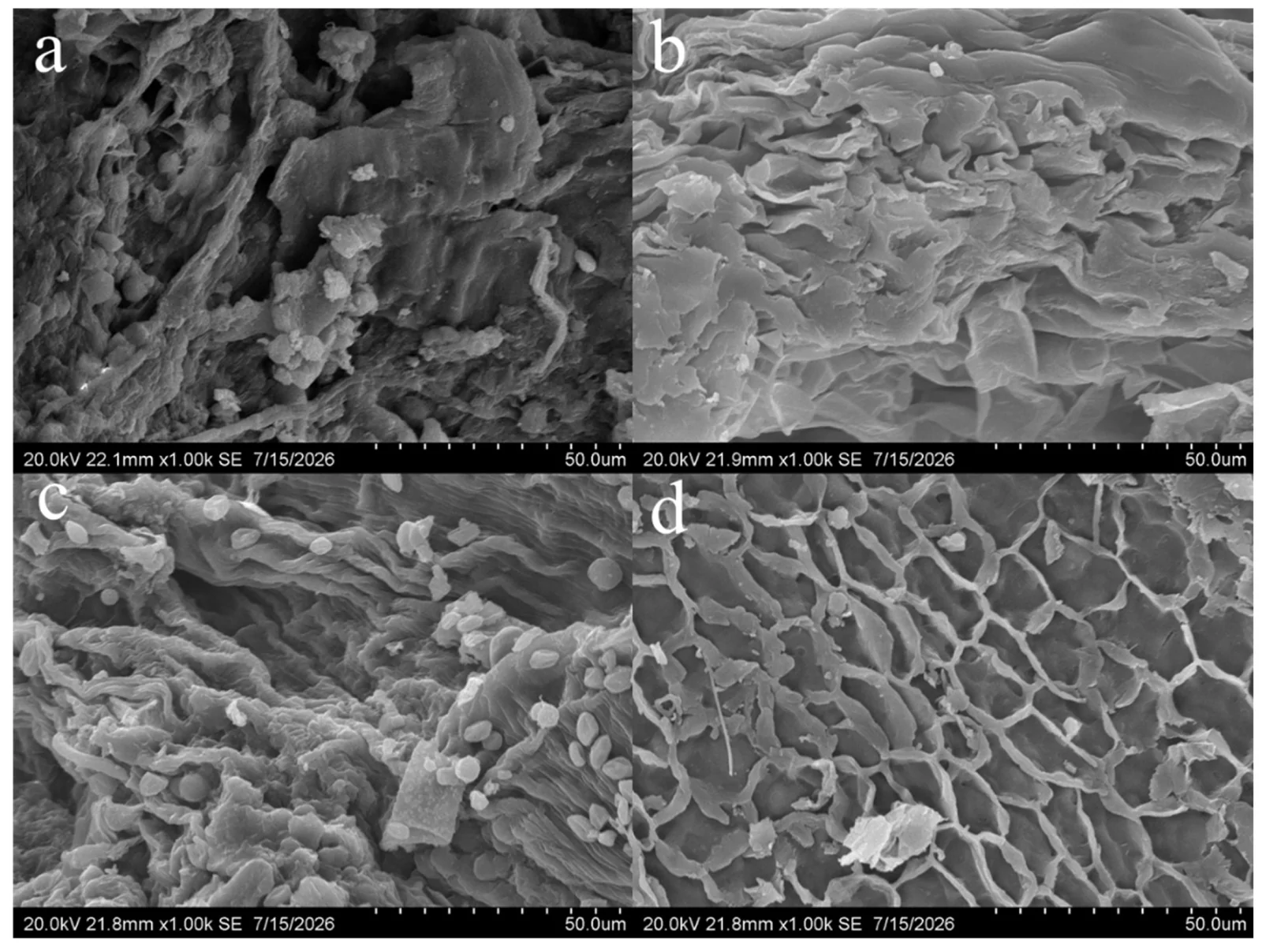
SEM of mulberry residues extracted with different PEG solutions: PEG 200 (**a**), PEG 300 (**b**), PEG 400 (**c**), and PEG 600 (**d**).

**Figure 5:**
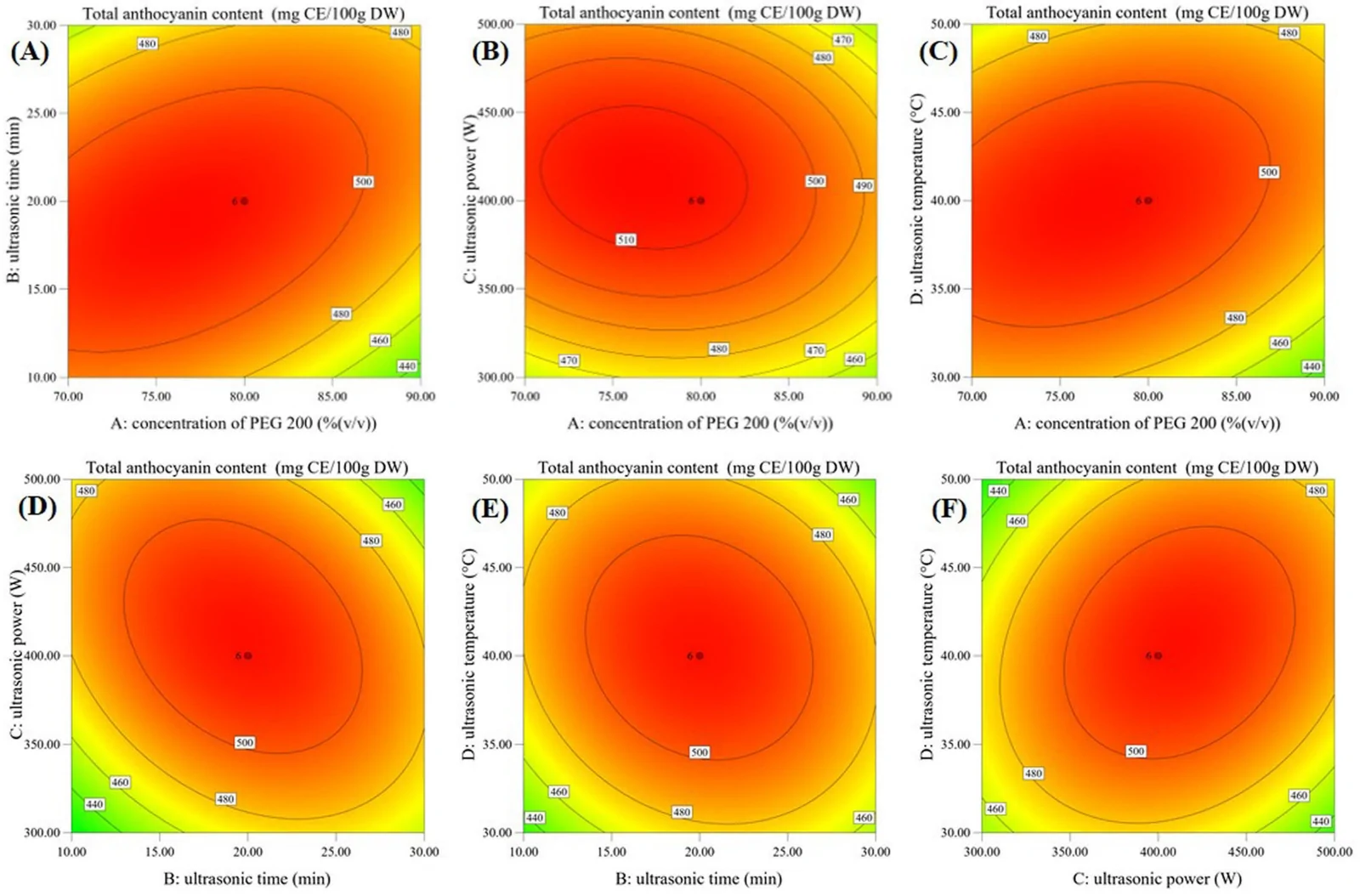
2D contour plots showing the effects of the tested parameters on TAC: (**A**) PEG 200 concentration and ultrasonic time, (**B**) PEG 200 concentration and ultrasonic power, (**C**) PEG 200 concentration and ultrasonic temperature, (**D**) ultrasonic time and ultrasonic power, (**E**) ultrasonic time and ultrasonic temperature, and (**F**) ultrasonic power and ultrasonic temperature. Different colors represent different predicted TAC values, as indicated by the color scale.

**Figure 6:**
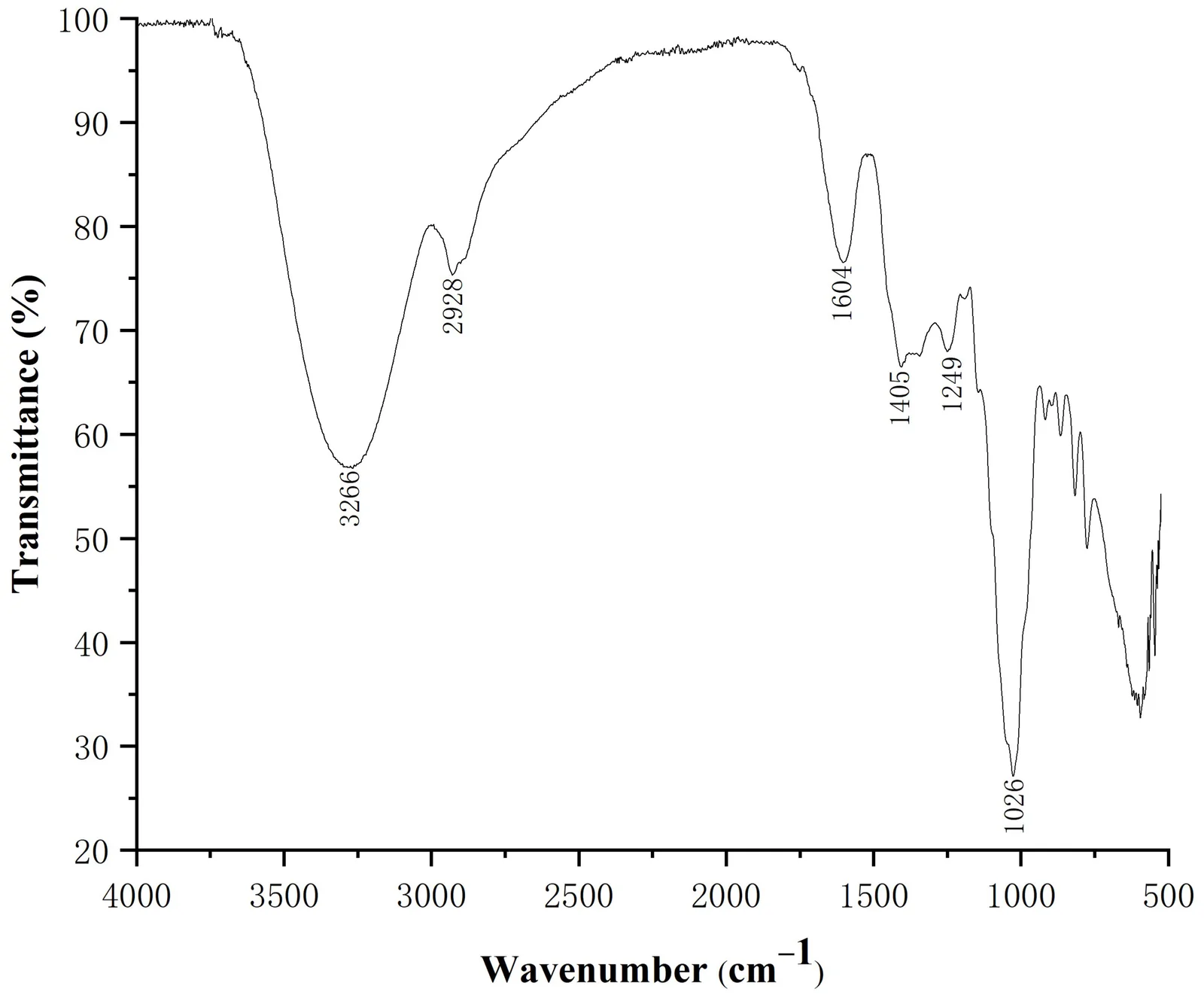
FT-IR spectrum of purified mulberry anthocyanin extract obtained by PEG200-assisted extraction and C18 SPE purification.

**Figure 7:**
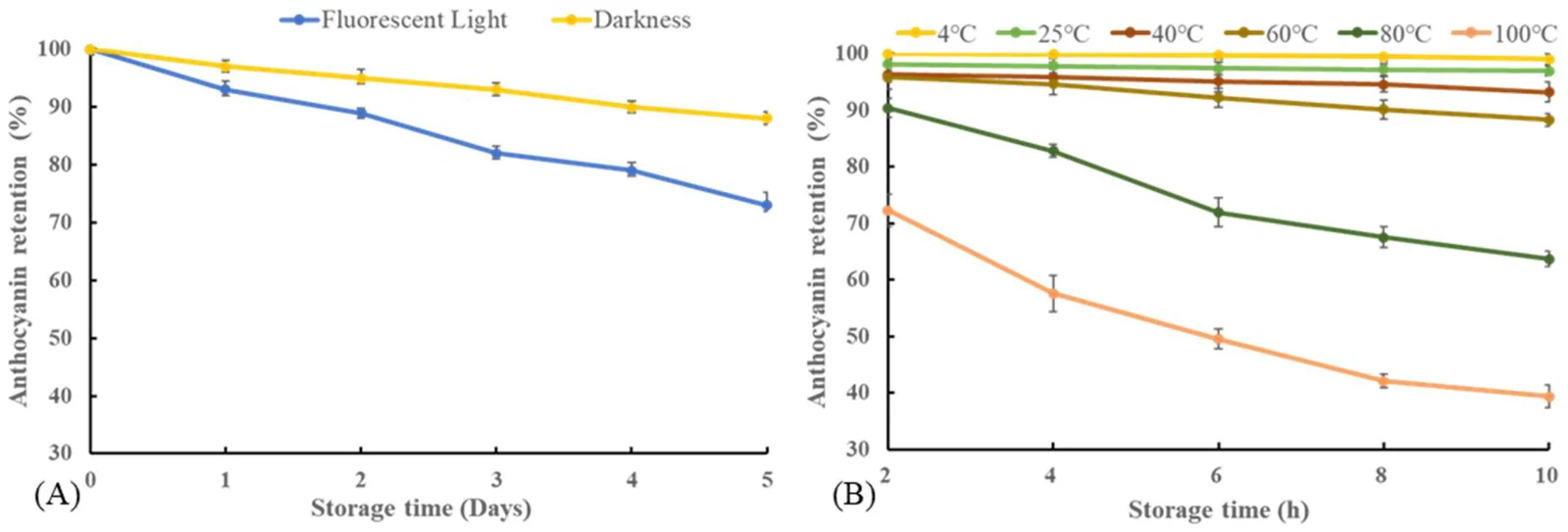
Stability of mulberry anthocyanins under light and temperature treatments. (**A**) Storage under fluorescent light and darkness. (**B**) Storage at different temperatures. Values are expressed as mean ± SD (*n* = 3).

**Table 1:** Response surface experiment design with the results.

Run	A: Concentration of PEG 200 (%, *v*/*v*)	B: Ultrasonic Time (Min)	C: Ultrasonic Power (W)	D: Ultrasonic Temperature (°C)	Experimental Values of TAC (mg CGE/100 g DW)	Predicted Values of TAC (mg CGE/100 g DW)
1	60	20	400	40	468.44	466.97
2	90	30	500	30	360.98	362.04
3	70	30	500	50	372.37	371.17
4	70	30	500	30	384.08	383.77
5	80	20	400	60	382.95	381.80
6	90	30	300	30	431.75	432.40
7	80	0	400	40	383.94	379.25
8	90	30	300	50	413.24	414.81
9	80	40	400	40	380.78	380.80
10	90	10	500	50	429.14	430.43
11	80	20	400	40	514.32	514.67
12	90	10	300	30	324.92	329.50
13	70	30	300	50	351.18	352.05
14	90	30	500	50	410.24	410.13
15	70	30	300	30	428.23	430.32
16	80	20	400	40	513.62	513.54
17	70	10	500	30	440.03	441.84
18	70	10	500	50	471.88	472.52
19	80	20	400	40	516.62	513.54
20	80	20	400	20	372.23	368.71
21	80	20	400	40	508.28	513.54
22	80	20	600	40	388.35	388.89
23	80	20	400	40	516.26	513.54
24	80	20	400	40	512.15	513.54
25	70	10	300	30	407.07	408.47
26	100	20	400	40	430.14	426.95
27	80	20	200	40	365.41	360.21
28	90	10	500	30	338.65	339.07
29	90	10	300	50	353.59	355.19
30	70	10	300	50	371.16	373.48

**Table 2:** ANOVA for the quadratic model of PEG200-assisted extraction of anthocyanins from mulberry.

Source	Sum of Squares	Degree of Freedom	Mean Square	*F*-Value	*p*-Value
Model	1.031 × 10^5^	14	7365.16	651.65	<0.0001
A—concentration of PEG 200	2401.08	1	2401.80	212.51	<0.0001
B—ultrasonic time	3.61	1	3.61	0.3195	0.5802
C—ultrasonic power	1234.24	1	1234.24	109.20	<0.0001
D—ultrasonic temperature	256.96	1	256.96	22.73	0.0002
AB	6568.70	1	6568.70	581.18	<0.0001
AC	566.56	1	566.56	50.13	<0.0001
AD	3682.37	1	3682.37	325.81	<0.0001
BC	6387.61	1	6387.61	565.16	<0.0001
BD	1872.94	1	1872.94	165.71	<0.0001
CD	4312.88	1	4312.88	381.59	<0.0001
A^2^	7600.11	1	7600.11	672.44	<0.0001
B^2^	30,558.72	1	30,558.72	2703.76	<0.0001
C^2^	33,118.74	1	33,118.25	2930.26	<0.0001
D^2^	32,781.25	1	32,781.25	2900.40	<0.0001
Residual	169.53	15	11.30		
Lack of Fit	122.44	10	12.24	1.30	0.4074
Pure Error	47.10	5	9.42		
Cor Total	1.033 × 10^5^	29			

The findings corroborate the suitability of the quadratic model developed through the Central Composite Design for optimizing anthocyanin extraction from Mulberry fruits. The *p*-values associated with factors A, C, D, and their interactions (AB, AC, AD, BC, BD, CD), as well as their quadratic terms (A^2^, B^2^, C^2^, D^2^), were all below the 0.05 threshold, indicating their significant influence on anthocyanin yield. To elucidate the interactions among variables and their impact on the response, six-dimensional response surface plots were generated (Figure 5).

**Table 3:** Identification of anthocyanins in purified mulberry extract by UHPLC-Q-TOF-MS/MS.

No.	RT (Min)	Compound	[M]^+^ (*m*/*z*)	MS^2^ (*m*/*z*)
1	4.358	Cyanidin hexose deoxyhexose hexoside	757	595; 449; 287
2	4.891	Cyanidin dihexoside	611	449; 287
3	7.319	Cyanidin 3-O-glucoside	449	287
4	7.980	Cyanidin 3-O-rutinoside	595	449; 287
5	8.180	Pelargonidin 3-O-glucoside	433	271

**Table 4:** Antibacterial effects of optimized PEG 200 extracts on bacteria.

Type	*E. coli*	*S. aureus*	*B. subtilis*
inhibition zone (mm)	12.16 ± 0.54	18.24 ± 0.82	19.28 ± 0.61

## Data Availability

The original contributions presented in this study are included in the article. Further inquiries can be directed to the corresponding authors.
